# Complications related to induced abortion: a combined retrospective and longitudinal follow-up study

**DOI:** 10.1186/s12905-018-0645-6

**Published:** 2018-09-25

**Authors:** Isabelle Carlsson, Karin Breding, P.-G. Larsson

**Affiliations:** 1 Skaraborg Hospital Skövde, Lövängsvägen, Skövde, Sweden; 2 Department of Obstetrics and Gynecology, Skaraborg Hospital Skövde, Skövde, Sweden; 3 Department of Clinical and Experimental Medicine, University of Linköping, Linköping, Sweden

**Keywords:** Induced abortion, Pregnancy, Endometritis, Vacuum curettage, Sexually transmitted diseases

## Abstract

**Background:**

Induced abortion is one of the most common gynecological procedures in Sweden, but there is still little knowledge about the adverse effects. The aims of this study are to provide an overview of complications of medical and surgical abortions and to evaluate the impact of bacterial screening to prevent postabortal infections.

**Methods:**

All women who underwent induced abortion at Skaraborg Hospital between 2008 and 2015 are included in the study. Bacterial screening for chlamydia, gonorrhea, mycoplasma, and bacterial vaginosis was performed prior to the abortions. Abortion complications, categorized as bleeding, infection, or incomplete abortion were assessed in women who came in contact with the gynecological clinic within 30 days after the procedure.

**Results:**

A total of 4945 induced abortions were performed during the study period. Nearly all, 4945 (99.7%) were eligible for inclusion in the study. Medical abortions < 12 weeks were the most common procedure (74.7%), followed by surgical abortions (17.5%), and medical abortion > 12 weeks (7.8%). Complications were registered in 333 (6.7%) of all abortions. Among medical abortions < 12 weeks, the complication frequency increased significantly, from 4.2% in 2008 to 8.2% in 2015 (RR 1.49, 95% 1.04–2.15). An incomplete abortion was the most common complication related to medical abortions < 12 weeks. Of all women who tested positive for one or several bacteria at the screening and therefore received antibiotics, 1.4% developed a postabortal infection. Among those who tested negative at the screening, 1.7% developed infectious complications.

**Conclusions:**

The share of complications related to medical abortions < 12 weeks increased significantly during 2008–2015 without any evident cause. Women who tested positive for one or several bacteria upon screening and received antibiotics experienced almost an equal proportion of postabortal infections compared to women who tested negative upon screening. The screening process seems to fulfill its purpose of reducing the risk of infectious complications.

## Background

An induced abortion is one of the most common gynecological procedures in Sweden. There were 36,600 induced abortions reported to The National Board of Health and Welfare (Socialstyrelsen) during 2014. The majority (93%) of which, were performed before the 12th week of pregnancy. The rate of abortions was 20 per 1000 women by the age of 15–44. The most common method was a medical abortion, which was used in 88% of all cases. [1] Early medical abortions (< 9 weeks of gestation) were introduced in Sweden 1992 when Mifepristone became available [2]. Before that, surgical abortions were the only legal option < 12 gestational weeks.

Despite well-developed abortion methods, there are known risks and adverse effects that must be considered. Potential complications related to abortions include pain, bleeding, an incomplete abortion, or an infection in the upper genital tract that causes endometritis, oophoritis, parametritis, and salpingitis. In Finland, Niinimäki et al. investigated the rate of complications related to medical abortions. Of the 24,006 adult participants who underwent a medical abortion, 15.4% were later diagnosed with bleeding, 2.0% experienced an infection, 10.2% experienced an incomplete abortion, and 13.0% had to proceed with a vacuum curettage [3].

Infections related to abortions are often caused by an ascending bacterial infection such as chlamydia, gonorrhea, mycoplasma and bacterial vaginosis (BV) that proceeds from the lower genitals and moves through the cervix to the uterus [4]. The infection, if untreated, can spread to the fallopian tubes and may lead to infertility.

The reported incidence of postabortal infections varies between studies, likely depending on local differences in diagnostics, the study population, laws and regulations, and the prevalence of sexually transmitted diseases. In Skaraborg, where the present study takes place, the number of postabortal infections was evaluated by Charonis and Larsson in 2006 [5]. They reported a frequency of 2.4% for infectious complications from medical abortions and 4.9% from surgical abortions. Another study conducted in Sweden and Norway investigated the infection rate after surgical abortions. They reported infections among 4.8% of the patients [6]. These patients had not received any prophylactic antibiotics. Several studies have shown that pre-abortion treatment for BV [6, 7] is effective in reducing the rate of postabortal infections.

The World Health organization (WHO) recommends antibiotic prophylaxis to prevent infectious complications associated with abortions. However, the WHO points out that there is only evidence for prophylactic antibiotics performing a surgical abortion when the risk of infections is more evident [8]. Additionally, antibiotic prophylaxis for all women undergoing abortion procedures likely results in overuse of antibiotics, which is associated with antibiotic resistance [9]. To address the problem of antibiotic overuse, it has been suggested that a screen and treat method may be more appropriate [10].

When using the screen and treat method, all patients undergoing abortion procedures will be tested for a number of selected bacteria. Antibiotic treatment is given if/when a bacterial infection is identified, which could be before, during, or after the abortion. Prior research indicated that the timing of antibiotic administration relative to the timing of the abortion does not affect the rate of postabortal infection [5–7]. The screen and treat strategy has several benefits. It enables targeted antibiotic treatment, which helps in reducing the use of antibiotics. This counteracts the negative aspects of antibiotic treatment, such as drug reactions and the development of antibiotic resistance among bacterial strains. The screening process also identifies patients with sexually transmitted diseases, which enables contact tracing. In that way, it has a positive impact on contamination and reinfection from sexual partners.

The main purpose of this study was to provide an overview of complications related to induced abortion and to investigate the difference in complications between medical and surgical abortions. The second purpose was to evaluate the impact of bacterial screening on the rate of postabortal infections.

## Methods

This is a combined retrospective and prospective follow-up study of women who had an induced abortion at Skaraborg Hospital between 2008 and 2015. From 2011 all complications are prospectively registered. Skaraborg Hospital consists of the hospitals in Skövde, Lidköping, Mariestad, and Falköping. Skaraborg Hospital system is the only provider of abortions in the area. The abortions were managed by the gynecological clinics at all four hospitals. Lidköping hospital was however not included until 2013 when it became part of Skaraborg Hospital. Before that it was a separate hospital that managed both medical and surgical abortions. Lidköping Hospital is located a 1 hour drive from Skövde Hospital.

Women considering an induced abortion were seen by a gynecologist at the gynecological clinic. The length of the pregnancy was established through a vaginal ultrasound by measuring the crown rump length (CRL). The gestational age was reported as weeks + days (7 + 3 etc.). The doctor also performed a gynecological examination, and samples were taken for bacterial screening. The bacterial screening program at Skaraborg Hospital included *C. trachomatis, N. gonorrhoeae, M. genitalium,* and bacterial vaginosis. Women who did not proceed with an abortion are not included in the study. For most women, medical abortion at home was the recommended procedure. Medical abortion completed in the hospital was recommended for women who were young, nulliparous, or more than 12 weeks gestation. Surgical abortion, using vacuum curettage, was performed if the women demanded this or had difficulties understanding the information about medical abortion. A surgical abortion, also called vacuum curettage, is allowed up to 12 + 0 gestational weeks but is not recommended before the 7th week because of the risk of an incomplete abortion.

A medical abortion < 12 weeks is initiated by giving the patient mifepristone (Mifegyne ®) 200 mg × 1 orally day one to stop the development of the pregnancy. The treatment continues on the third day with misoprostol (Cytotec ®) 0.2 mg × 4 vaginally, which will help the uterus contract and expel the pregnancy. A medical abortion that is performed after 12 gestational weeks (12 + 1) takes place at the hospital. The protocol is the same with mifepristone and the misoprostol first 4 vaginally and then 2 orally every 3rd hour until abortion. Medical abortions > 12 weeks are often completed by a vacuum curettage as part of the normal course. A vacuum curettage is in these cases not counted as treatment for a complication.

Women who had medical abortions < 12 weeks were informed that a woman should expect heavy bleeding 4–12 h after taking of the misoprostol and then bleeding similar to a heavy period for the next 2–5 days. Women who had a surgical abortion were informed to expect a bleeding for 1 week. All women were scheduled for a follow-up visit with a midwife approximately 1 month after the abortion. The main purpose of the follow-up visit was to ensure that the pregnancy had been terminated properly by analyzing the levels of human chorionic gonadotropin hormone in the urine and to discuss contraceptive methods. Complications in the other hand were always handled by a doctor. Women were encouraged to contact one of the gynecological clinics at Skaraborg Hospital for gynecological evaluation with a doctor if they experienced significant bleeding after 7 days, abdominal pains, fever or malodorous discharge/bleeding after the abortion.

During 2008–2010, abortion records were collected retrospectively. Data collected included the number of previous induced and spontaneous abortions, parity, length of current pregnancy, results of bacterial screening, prescribed antibiotic treatment, and the chosen abortion method. Women were followed through the patient records system to determine the rate of complications. All patients who had a visit with a doctor at the gynaecological clinic within 30 days after the abortion were included. The cause of the visit was determined through review of patient records and entered into the spreadsheet as an infection, bleeding, incomplete abortion, or other (e.g contraceptive consulting (not a complication)).

From 2011 to 2015, data from the abortion complications were collected prospectively by an administrator at the gynecological clinic. If a patient visited the gynecological clinic within two calendar months of the abortion, paper copies of the patient records were made and then given to one of the gynecologists. The gynecologist evaluated the time and the cause of the visit (infection, bleeding, incomplete abortion, or other) by reading the patient records and reported back so that complications within 30 days from the abortion could be recorded.

Skaraborgs hospital is located in a rural area. As such, there are very few alternatives to seek for medical treatment except going back to the hospital. The hospital has three outpatient clinics using the same computer medical record.

### Outcome definitions

An infection in this context is referred to as postabortal endometritis, which is an inflammation in the uterus caused by a bacterial infection. To be categorized as an infection, a patient must have had symptoms of infection and received antibiotics from a doctor. The symptoms may be bleeding or discharge, lower abdominal pains, a fever, and/or an elevated CRP (C-reactive protein). An infection can sometimes be caused by an incomplete abortion. If an infection and an incomplete abortion occurred at the same time, it was categorized as an incomplete abortion.

Bleeding is often a part of the normal course of an abortion. Only heavy bleeding lasting longer than 12 h or longstanding bleeding (> 21 days) was categorized as a bleeding complication. The diagnosis of bleeding complications was set by the doctor when the patients came to the gynecological department. Furthermore, to categorize the complication as bleeding, the patient could not have had a simultaneous infection or incomplete abortion.

If the vaginal ultrasound revealed uterine contents of 15 mm or more, resembling placental tissue, treatment with mifepristone was performed. If the bleeding stopped with this treatment, it was classified as a bleeding complication. However, if any significant evidence of retained products of conception were detected on ultrasound scan, vacuum curettage was scheduled and the complication was defined as an incomplete abortion. Women with an incomplete abortion and simultaneous signs of infection were counted as incomplete abortions since incomplete abortions are a natural cause of infections.

In abortions, after 12th week of gestation, the remaining placental tissue can be treated with extra mifepristone or vacuum curettage. However, this is not considered a complication if this is done during the hospital visit. If the patients return after discharge from the hospital with retained placental tissue, this is regarded as an incomplete abortion complication.

### Statistical analysis

The data were described using frequencies and proportions. SPSS version 22 was used for calculating a relative risk with a 95% confidence interval.

## Results

A total of 4945 induced abortions were performed during the study period. Sixteen patients were excluded, resulting in 4945 induced abortions included in the study. The mean age of the women receiving abortions was 26 years and half were nulliparous (53.9%). The majority of women (63.5%) had not had a prior induced abortion (range 0–6 induced abortions).

**Fig. 1:**
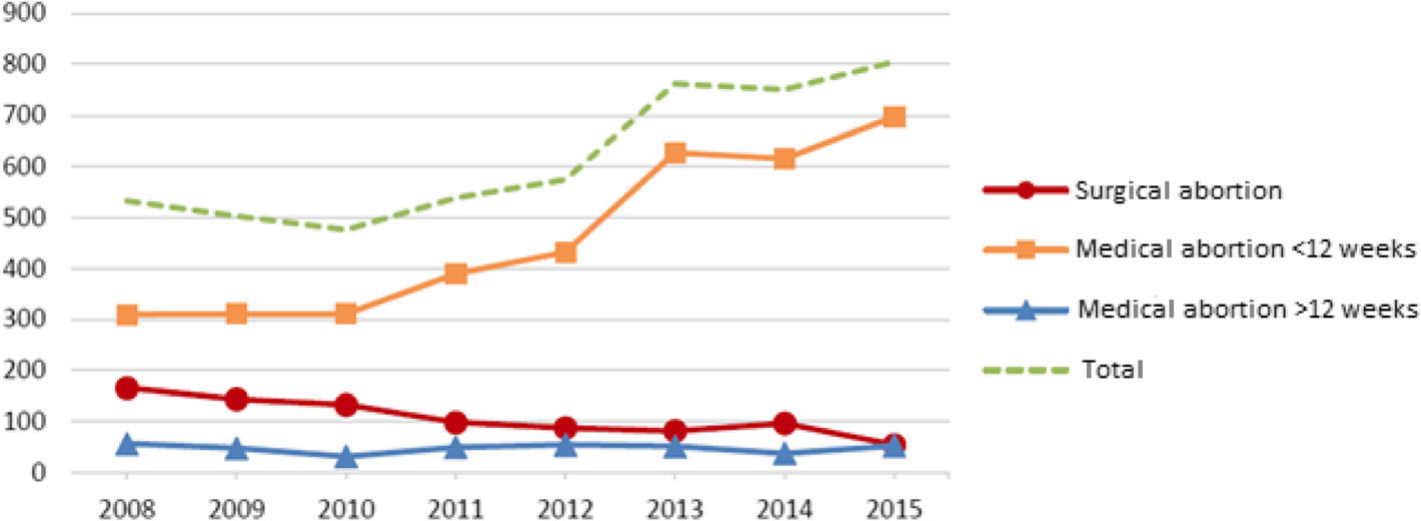
Number of induced abortions per year from 2008 to 2015 at Skaraborg Hospital. The total number of abortions per year is illustrated together with the three different abortion methods: medical abortion > 12 weeks, medical abortion < 12 weeks and surgical abortion. Data from 2008 to 2012 include the clinics in Skövde, Falköping, and Mariestad. Lidköping’s hospital was added to the statistics in 2013

The number of induced abortions gradually increased throughout the study, from 533 to 805 abortions per year (Fig. 1; Table 1). Medical abortions < 12 weeks were the most frequently used method. The share of medical abortions < 12 weeks increased from 58.2 to 86.6% between 2008 and 2015 while the share of surgical abortions (31.1% vs 6.8%) and medical abortions > 12 weeks (10.7% vs 6.6%) has decreased.

**Table 1:** All induced abortions at Skaraborg Hospital in 2008–2015. This table shows the number and share of induced abortions performed by the three abortion methods

	2008	2009	2010	2011	2012	2013	2014	2015	Total
	n (%)								
Medical Abortions < 12 weeks	310 (58.2)	311 (61.8)	311 (65.3)	390 (72.4)	432 (75.1)	628 (82.4)	617 (82.0)	697 (86.6)	3696 (74.7)
Surgical Abortions	166 (31.1)	144 (28.6)	133 (27.9)	99 (18.4)	88 (15.3)	82 (10.8)	97 (12.9)	55 (6.8)	864 (17.5)
Medical Abortions > 12 weeks	57 (10.7)	48 (9.5)	32 (6.7)	50 (9.3)	55 (9.6)	52 (6.8)	38 (5.1)	53 (6.6)	385 (7.8)
Total Abortions	533 (10.8)	503 (10.2)	476 (9.6)	539 (10.9)	575 (11.6)	762 (15.4)	752 (15.2)	805 (16.3)	4945

**Fig. 2:**
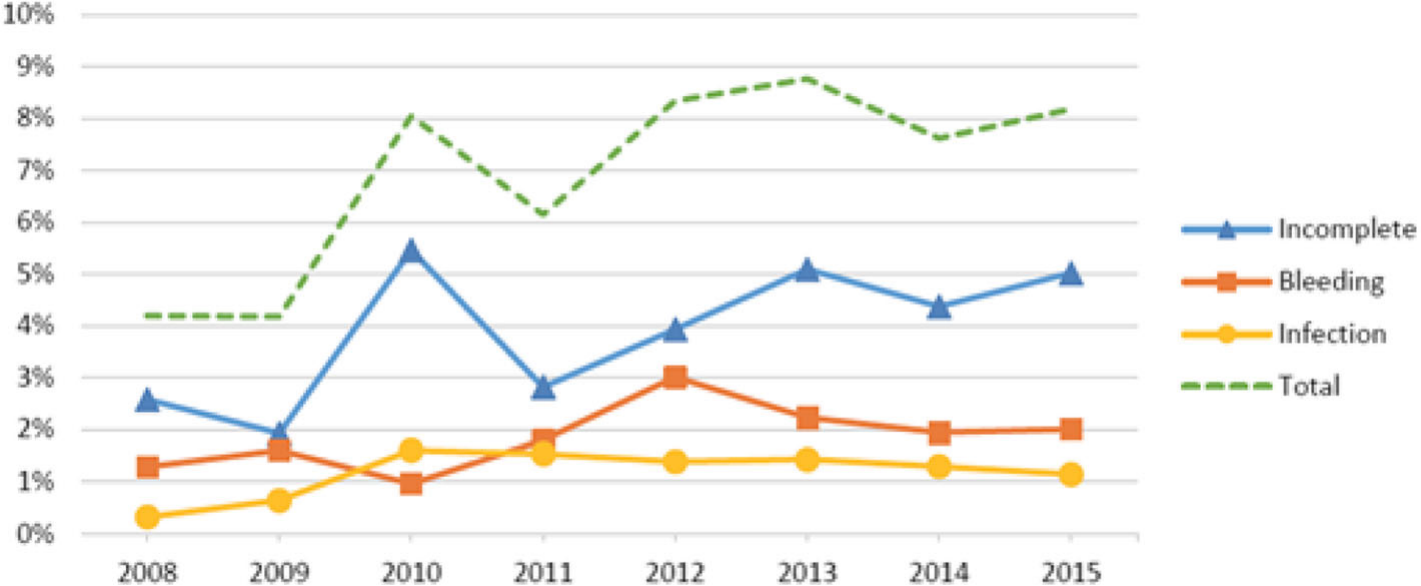
Complication frequency among medical abortions < 12 weeks in Skaraborg i 2008–2015. The diagram shows separate graphs for incomplete abortions, bleeding, infections, and the total complication frequency

Over the course of the study, complications occurred in 6.7% (*n* = 333) of all induced abortions. Table 2 presents data on postabortal complications by method. Complications occurred in 7.3% of the medical abortions < 12 weeks. Over time, the rate of complications for medical abortions < 12 weeks increased from 4.2% in 2008 to 8.2% in 2015 (Fig. 2). This difference was significant (RR 1.49, 95% 1.04–2.15). The most common complication (57%) related to medical abortions < 12 weeks was incomplete abortions, which occurred 153 times, comprising 4.1% of all medical abortions. The frequency of infections related to medical abortions < 12 weeks was only 1.2%.

**Table 2:** The relative number and share of complications in each abortion method between 2008 and 2015. The complications are categorized as incomplete abortion, bleeding or infection

	2008	2009	2010	2011	2012	2013	2014	2015	Total
	n (%)								
Complications following Medical Abortions < 12 weeks	13 (4.2)	13 (4.2)	25 (8.0)	24 (6.2)	36 (8.3)	55 (8.8)	47 (7.6)	57 (8.2)	270 (7.3)
Incomplete Abortions	8	6	17	11	17	32	27	35	153
Bleeding	4	5	3	7	13	14	12	14	72
Infections	1	2	5	6	6	9	8	8	45
Complications following Surgical Abortions	11 (6.6%)	6 (4.2%)	4 (3.0%)	7 7.1%)	7 (8.0%)	6 (7.3%)	2 (2.1%)	2 (3.6%)	45 (5.2%)
Incomplete Abortions	5	3	1	0	2	2	1	1	15
Bleeding	0	0	0	2	1	1	0	0	4
Infections	6	3	3	5	4	3	1	1	26
Complications following Medical Abortions > 12 weeks	2 (3.5%)	4 (8.3%)	2 (6.3%)	3 (6.0%)	2 (3.6%)	1 (1.9%)	3 (7.9%)	1 (1.9%)	18 (4.7%)
Incomplete Abortions	0	0	1	2	2	0	3	1	9
Bleeding	0	1	1	1	0	0	0	0	3
Infections	2	3	0	0	0	1	0	0	6

**Fig. 3:**
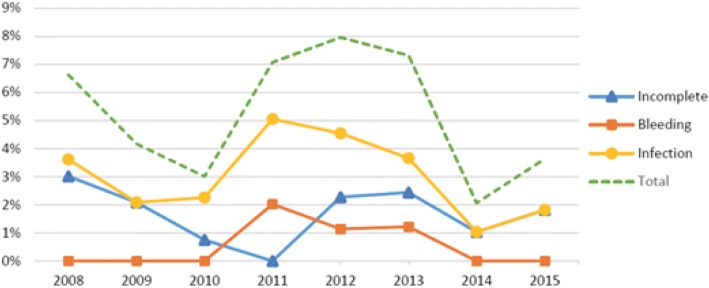
Complication frequency among surgical abortions in Skaraborg in 2008–2015. The diagram shows separate graphs for incomplete abortions, bleeding, infections, and the total complication frequency

There were 864 surgical abortions and 45 cases of complications, which gives a total complication frequency of 5.2% (Table 2; Fig. 3). There was an increase in the share of complications during 2011–2013 with a maximum of 8.0% during 2012. Infections were the most common complications related to surgical abortions, representing more than half of the postoperative complications. Bleeding was a rare complication; only 4 cases of abnormal bleeding following surgical abortions were observed. Only 385 abortions were performed after the 12th week during 2008–2015. In 18 of them, there were complications afterwards, which represent a complication frequency of 4.7%. Nine were incomplete abortions, six were infections, and three were bleeding.

Since there was a significant increase in complications related to medical abortions < 12 weeks, a comparison was made to determine whether there is any evident difference between patients undergoing abortion in 2008–2010 and 2015. The distribution of medical abortions by gestational weeks was examined. There was an increase in the proportion of medical abortions < 7 + 0 gestational weeks from 51.2% in 2008–2010 to 57.1% in 2015. The number of medical abortions at 9–12 gestational weeks also increased from 0.2% in 2008–2010 to 7.9% in 2015.

Table 3 compares the proportion of complications related to medical abortions during different gestational weeks. The rate of complications rose in abortions performed before the 7th week (RR 1.21 (95% CI 0.71–2.06) and between the 7th and 9th weeks (RR 1.62 (95% CI 0.94–2.78), but no one of these increases are significant separately. The share of medical abortions performed at home was also calculated. During 2008–2010, 74.6% of all medical abortions before the 9th gestational week were carried out at home compared with 85.2% during 2015. This is an increase of just over 10%.

**Table 3:** Complication frequency related to medical abortions < 12 weeks separated by different gestational weeks. Comparison between 2008 and 2010 and 2015

Gestational weeks	2008–2010	2015
Total	50/931 (5.4%)	57/697 (8.2%)
< 7 + 0	25/477 (5.2%)	27/398 (6.8%)
7 + 1–9 + 0	25/452 (5.5%)	24/244 (9.8%)
9 + 1–12 + 0	0/2 (0.0%)	6/55 (10.9%)

All medical abortions < 9 weeks during 2015 were separated by the location of the abortion (Table 4). Medical abortions at the hospital between 9 and 12 gestational weeks were not included in this calculation because home abortions are not an option after 9th week. The purpose was to investigate whether there was any difference in the number of complications between medical abortions performed at home or at the hospital. The complication frequency was significantly higher among women < 7 gestational weeks who had their abortions at home (RR 2.99 (95% CI 0.42–21.4)).

**Table 4:** Complications related to medical abortions < 12 weeks during 2015 separated by the different gestational weeks and the location of the abortion. The relative number of abortions and complications are presented in each group together with the complication frequency. The complication frequency was higher among women who had their abortions at home, but the difference was not significant (RR 1.30, 95% 0.57–2.97)

Gestational weeks	At home	At the hospital
Total	45/547 (8.2%)	12/150 (8.0%)
< 7	26/357 (7.3%)	1/41 (2.4%)
7 + 1–9 + 0	19/190 (10.0%)	5/54 (9.3%)
9 + 1–12 + 0	–	6/55 (10.9%)

**Fig. 4:**
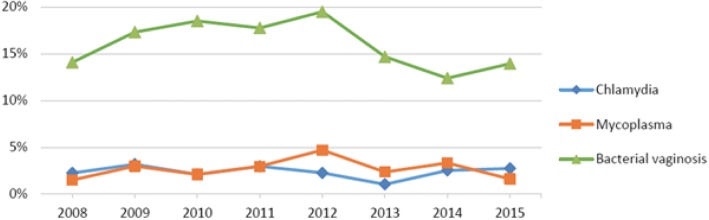
Prevalence of chlamydia, mycoplasma, and bacterial vaginosis upon bacterial screening

The prevalence of chlamydia, mycoplasma, and bacterial vaginosis is illustrated in Fig. 4. The bacterial screening detected 116 chlamydial infections during 2008–2015. The prevalence of chlamydia among women undergoing abortions varied between 1.0–3.0% per year. *M. genitalium* was found in 132 cases and had a total prevalence of 2.7%. Bacterial vaginosis was the most common infection, with 775 cases. The prevalence of bacterial vaginosis varied between 12.4–19.5% per year. Only one patient was diagnosed with gonorrhea during the study and therefore is not included in the figure.

The presence of coinfections was investigated in data collected from 2008 to 2010. A correlation was found between bacterial vaginosis and chlamydia. Among patients infected with chlamydia, 24.3% also had bacterial vaginosis (RR 1.56 (95% CI 1.12–2.17)). Among those who tested negative for chlamydia, 16.5% had bacterial vaginosis. An even stronger correlation was found between bacterial vaginosis and mycoplasma. Of all patients who tested positive for mycoplasma, 37.5% had bacterial vaginosis compared with 16.0% among women without mycoplasma (RR 2.46 (95% CI 1.95–3.10)). No correlation was found between chlamydia and mycoplasma. They affected the same age group but different individuals.

Of all women who tested positive for one or several bacteria at the screening and therefore received antibiotics, 1.4% developed a postabortal infection. Among those who tested negative at the screening 1.7% developed infectious complications. When including only surgical abortions, 1.1% had an infection after testing positive at the screening. Among those who did not have a bacterial infection upon screening, 3.2% developed a postabortal infection. This difference was not statistically significant (OR 0.330, 95% 0.042–2.592).

### Excluded patients

Of all 4961 induced abortions, 16 were excluded from the study. Six were failed medical abortions in which the pregnancy had proceeded. Four patients were excluded since they underwent diagnostic laparoscopy for suspected ectopic pregnancy at the same time as a vacuum curettage. One patient was excluded because she did not contact the gynecological clinic until the 38th day after her abortion. She was later diagnosed with an incomplete abortion and had a vacuum curettage. One patient terminated her pregnancy by a hysterectomy and was therefore excluded. This patient had been treated earlier with endometrial ablation for menorrhagia. She became pregnant after several years of amenorrhea, and the pregnancy was not discovered until the 18th week.

One patient was excluded because the surgical abortion had to be discontinued. The reason was that a false passage to the uterus had been created when dilating the cervix. Subsequently, the pregnancy was terminated successfully by a surgical abortion. One patient was excluded because of an interstitial pregnancy close to the fallopian tube. The patient had an incomplete medical abortion with a subsequent vacuum curettage. Despite this, she had abdominal pains and bleeding. When the interstitial pregnancy was finally discovered, the patient was treated with methotrexate and later, a subtotal hysterectomy. One patient was excluded because her personal code number could not be found in the patient records system. Another patient was excluded since there no information could be found on the length of her pregnancy, her number of previous abortions, and parities.

## Discussion

The first aim was to investigate the number of complications associated with induced abortions. The main finding after compiling the results was an increasing number of complications after medical abortions < 12 weeks that was statistically significant. Incomplete abortions were found to be the most common complication after medical abortions < 12 weeks. Concerning medical abortions > 12 weeks and surgical abortions, it has been difficult to discern whether there are any trends since the numbers of medical abortions > 12 weeks and surgical abortions are low and the cases of complications are few. The second aim was to evaluate the impact of bacterial screening on postabortal infections. The frequency of infections after induced abortions appeared to be equal or even lower among patients who had a bacterial infection upon screening and therefore did receive antibiotics. Despite bacterial screening, there were still patients who suffered from infectious complications.

When looking at the number of abortions each year (Fig. 1, Table 1), we can see an increase from 2008 when 533 abortions were performed, to 2015 when there were 805 abortions. This increase can primarily be explained by the addition of abortions being performed at Lidköpings Hospital from 2013 and forward. But this may not be the only reason. An increase in the number of abortions could be expected at this time because of the high number of children that were born in the early 1990’s [11]. Girls born during that decade should have reached an age of 20–24 years by now, and this is the age where the proportion of induced abortions is the highest. According to a statistical report from The National Board of Health and Welfare [1], the proportion of medical abortions has increased the last 15 years, while the proportion of surgical abortions has decreased. This matches our findings in Fig. 1 and Table 1.

There was a significant increase in the share of complications related to medical abortions < 12 weeks (RR 1.49, 95% 1.04–2.15). One potential reason is that the proportion of induced abortions performed at home has risen. It is likely that women who have medical abortions at home will visit our outpatient clinic in a greater extent since they do not have the direct help and support from a midwife. Later, they may have been diagnosed with a postabortal complication even though their symptoms are mild and could have resolved without any treatment. Table 4 shows that abortions at home are associated with a greater proportion of complications, but this is not statistically significant. This is likely due to of the low number of abortions performed at the hospital.

The number of infections after medical abortions < 12 weeks is 1.2%, which is low compared with Charonis et al.’s (2006) reported frequency of 2.4% [5]. The actual number of infections after medical abortions < 12 weeks in our study is, however, low: only 45 patients in 8 years, which makes the results difficult to validate. As many as 4.1% of the medical abortions < 12 weeks had to be supplemented by a vacuum curettage.

There were also six failed medical abortions that were excluded from the statistics (see excluded cases). A failed abortion must be interpreted as the worst kind of incomplete abortion. When reading the patient records, it was evident that many patients did not attend the follow-up visits. It is of utmost importance to explain to the patients why this appointment is so essential and to motivate them to come.

There were few surgical abortions compared to medical abortions, and the relative number of complications related to surgical abortions was low; most were infections, which we expected. When performing a vacuum curettage, there is always a risk that bacteria from the lower genitals will be brought up to the uterus, causing endometritis. Figure 3 shows a peak in the complication frequency during 2011–2013. However, according to Table 2, this increase only consists of a few more cases, so it could be just a coincidence. The total complication frequency after surgical abortions was 5.2%, which is consistent with previous research [5].

The prevalence of chlamydia among women undergoing induced abortions was consistent with previous research. One of the latest studies on the subject from Sweden by Bjartling et al. (2010) reported a prevalence of chlamydia of 2.8% compared to our average of 2.3% [4]. Mycoplasma was present in 2.7% of all the patients, which was nearly the same as Bjartling et al. (2010) (2.5%). The prevalence of gonorrhea was very low, as expected. Only one patient had gonorrhea, which is 0.02% of all patients. Bacterial vaginosis seems to be less prevalent in this study compared with others. The prevalence of bacterial vaginosis was 15.7%, compared with approximately 20% in two other Swedish studies [5, 6].

A comparison was made between all patients who tested positive for one or several bacteria upon screening and received antibiotics and those whose tests were negative. We found that the share of infectious complications was almost the same among patients who had tested positive at the screening. The screen and treat policy, where patients have to wait for the screening results before treatment does not appear to increase the risk and might be a better choice than antibiotic prophylaxis for all patients.

When including only surgical abortions, there was a greater difference between the two groups. There were fewer infectious complications among patients treated with antibiotics for chlamydia, gonorrhea, mycoplasma, or bacterial vaginosis. This indicates that antibiotics may have a positive impact, beyond treating the screened infections. There was, however, no significant difference between these groups, probably because of the low number of surgical abortions.

It is, however, clear that bacterial screening cannot prevent all cases of postabortal infections. There must be other bacteria involved, beyond those that are screened for. In a study from 2009, the presence of pathogens in cervical samples among 114 women with upper genital tract infections was investigated [12]. It showed that bacteria from the upper respiratory tract were detected more times than sexually transmitted bacteria. *Haemophilus influenza*, Group A streptococci, and *Streptococcus pneumoniae* were detected in nine cases compared with *C. trachomatis* and *N. gonorrhoeae*, which were identified in seven of the patients. Perhaps bacteria from the upper respiratory tract are involved in developing postabortal infections or increasing bacterial resistance could play a role.

This study has several limitations. The timeline of 30 days regarding the follow-up of the patients proved to have disadvantages. Some patients did not contact the gynecological clinic for their postabortal problems until after 30 days, which leads to an underestimation in the amount of complications. One limitation is that some patients may be missing in our statistics since they sought medical help somewhere other than at Skaraborg Hospital. However, there are few private gynecological clinics in the area, so it can be assumed that the majority of the complications are represented in this material.

Another limitation is that antibiotic treatment for the screened infections could not always be given prior to the abortion since the analysis for chlamydia, gonorrhea, and mycoplasma takes 2 days. This means that antibiotics were sometimes given one or 2 days after the day of the abortions. This dilemma primarily affects medical abortions, which are often initiated the same day as the appointment at the gynecologist. Surgical abortions are usually performed after a week or so, which facilitates the timing of the treatment. According to a study by Larsson et al. [7], the time from abortion to infection is usually four to 5 days.

Another limitation is that various individuals were involved in categorizing the complications. This may have resulted in misdiagnosis. In other words, patients who appeared to be similar to each other might have been categorized differently.

One of the strengths in this study is the size of the study group. A total of 4945 induced abortions were included, which gave a fairly accurate depiction of the incidence of bacterial infections in the screening process and of the overall complication frequency.

## Conclusions

The rate of complications associated with medical abortions < 12 weeks has increased from 4.2% in 2008 to 8.2% in 2015. The cause of this is unknown but it may be associated with a shift from hospital to home medical abortions. Women who tested positive for one or several bacteria upon screening and received antibiotics had almost an equal proportion of postabortal infections as women who tested negative upon screening. The screening process seems to fulfill its purpose of reducing the risk of infectious complications. However, the use of bacterial screening did not eliminate all cases of postabortal infections. There are likely other bacteria involved that we do not know about yet.

## Abbreviations

BV: Bacterial vaginosis
CI: Confidence interval
CRL: Crown rump length
EPN: Regional ethical review board
PCR: Poly chain reaction
RR: Relative risk
WHO: World Health Organization
